# Feasibility of antiretroviral treatment monitoring in the era of decentralized HIV care: a systematic review

**DOI:** 10.1186/s12981-017-0131-5

**Published:** 2017-01-19

**Authors:** Minh D. Pham, Lorena Romero, Bruce Parnell, David A. Anderson, Suzanne M. Crowe, Stanley Luchters

**Affiliations:** 1 Burnet Institute, 85 Commercial Road, Melbourne, VIC 3004 Australia; 2 Department of Epidemiology and Preventive Medicine, Faculty of Medicine Nursing and Health Science, Monash University, Melbourne, Australia; 3 The Alfred Hospital, The Ian Potter Library, Melbourne, VIC Australia; 4 Department of Microbiology, Faculty of Medicine Nursing and Health Science, Monash University, Melbourne, Australia; 5 Department of Infectious Diseases, The Alfred Hospital Melbourne, Melbourne, Australia; 6 Monash School of Medicine, Faculty of Medicine Nursing and Health Science, Monash University, Melbourne, Australia; 7 International Centre for Reproductive Health, Department of Obstetrics and Gynecology, Faculty of Medicine and Health Sciences, Ghent University, Ghent, Belgium

**Keywords:** HIV, Decentralized care, Task-shifting, Antiretroviral treatment, Treatment monitoring, Viral load, CD4, Systematic review

## Abstract

**Background:**

Regular monitoring of HIV patients who are receiving antiretroviral therapy (ART) is required to ensure patient benefits and the long-term effectiveness and sustainability of ART programs. Prompted by WHO recommendations for expansion and decentralization of HIV treatment and care in low and middle income countries, we conducted a systematic review to assess the feasibility of treatment monitoring in these settings.

**Methods:**

A comprehensive search strategy was developed using a combination of MeSH and free text terms relevant to HIV treatment and care, health service delivery, health service accessibility, decentralization and other relevant terms. Five electronic databases and two conference websites were searched to identify relevant studies conducted in LMICs, published in English between Jan 2006 and Dec 2015. Outcomes of interest included the proportion of patients who received treatment monitoring and health system factors related to monitoring of patients on ART under decentralized HIV service delivery models.

**Results:**

From 5363 records retrieved, twenty studies were included in the review; all but one was conducted in sub-Saharan African countries. The majority of studies (15/20) had relatively short follow-up duration (≤24 months), and only two studies were specifically designed to assess treatment monitoring practices. The most frequently studied follow-up period was 12 months and a wide range of treatment monitoring coverage was observed. The reported proportions of patients on ART who received CD4 monitoring ranged from very low (6%; N = 2145) to very high (95%; N = 488). The median uptake of viral load monitoring was 86% with studies in program settings reporting coverage as low as 14%. Overall, the longer the follow-up period, the lower the proportion of patients who received regular monitoring tests; and programs in rural areas reported low coverage of laboratory monitoring. Moreover, uptake in the context of research had significantly better where monitoring was done by dedicated research staff. In the absence of point of care (POC) testing, the limited capacity for blood sample transportation between clinic and laboratory and poor quality of nursing staff were identified as a major barrier for treatment monitoring practice.

**Conclusions:**

There is a paucity of data on the uptake of treatment monitoring, particularly with longer-term follow-up. Wide variation in access to both virological and immunological regular monitoring was observed, with some clinics in well-resourced settings supported by external donors achieving high coverage. The feasibility of treatment monitoring, particularly in decentralized settings of HIV treatment and care may thus be of concern and requires further study. Significant investment in POC diagnostic technologies and, improving the quality of and training for nursing staff is required to ensure effective scale up of ART programs towards the targets of 90-90-90 by the year 2020.

**Electronic supplementary material:**

The online version of this article (doi:10.1186/s12981-017-0131-5) contains supplementary material, which is available to authorized users.

## Background

Increasing access to antiretroviral therapy (ART) for people living with HIV/AIDS has been identified as a key strategy to curb the HIV epidemic and avoid its cost in the future [1]. In 2015, an estimated 15 million people living with HIV/AIDS (PLWHs) were receiving ART, a remarkable milestone in the fight against HIV/AIDS [2]. However, in order to achieve the ambitious sustainable development goal of ending the HIV epidemic by 2030, greater efforts are required in expanding ART coverage and improving quality of services with innovative and effective service delivery models.

In a number of the low and middle income countries (LMICs) most affected by the epidemic, decentralization of HIV treatment and care, linked with task-shifting, has been implemented in response to the need for scaling up service provision [3]. Evidence from existing systematic reviews suggests that relocation of ART services closer to patients’ homes through decentralized care can improve patient access and adherence to HIV treatment with non-inferior quality of care as compared to centralized, hospital-based care [4–6].

Current WHO guidelines on the use of ARV drugs for HIV treatment and prevention strongly recommend virological monitoring as the strategy of choice for monitoring responses to ART [7]. Immunological monitoring (CD4 testing) is being scaled back for assessment of treatment responses where VL testing is available, but will still be required for the foreseeable future in many settings to determine the level of HIV-induced immune deficiency, including the need for screening and prophylaxis for serious co-infections, and to prioritize initiation of HIV treatment. Clinical monitoring is essential for all patients who are receiving ART to monitor patient responses to treatment and diagnose potential treatment failure [8]. In addition, monitoring of ARV drug toxicity is recommended, as delaying drug substitutions when there are adverse drug effects may not only cause harm but also result in non-adherence leading to drug resistance and treatment failure. The latter will compromise the effectiveness of available ART regimens, increase spread of drug-resistant HIV, increase HIV incidence, morbidity and mortality and negatively impact the long-term sustainability and efficacy of ART programs in LMICs.

Given the current limited health system capacity in many LMICs, meeting WHO’s recommendations regarding regular monitoring of patients’ responses to treatment, including monitoring of drug toxicity, may pose major challenges to the health system with possible negative impacts on quality and sustainability of HIV services in the future [9, 10]. This vulnerable situation is particularly likely while rapid scale up of decentralized provision of ART is being prioritized.

This systematic review assessed the feasibility of ART treatment monitoring in settings of decentralized HIV treatment and care in LMICs.

## Methods

### Literature search strategy

The preferred reporting item for systematic reviews and meta-analysis (PRISMA statement) [11] was used to guide the conduct of this review. A literature search strategy was developed to identify relevant studies that involve decentralization of HIV treatment and care in low and middle income countries, published in English between Jan 2006 and Dec 2015. Key search terms include MeSH and free text terms relevant to HIV infection, HIV treatment and care, health service delivery models and service accessibility such as: “HIV”, “HIV infection”, “Antiretroviral therapy”, “ART”, “HAART”, “delivery of health care” “primary health care”, “community health services”, “home-based*”, “decentral*”, “task-shift*”. Search terms also included those that refer to treatment monitoring including “treatment outcomes”, “adverse effect” and “toxicity”. The search strategy was first conducted in Medline (see Additional file 1: Annex S1), then adapted to run across CENTRAL, CINAHL, EMBASE, Scopus and Web of science. Conference abstracts were also searched from International AIDS Society and CROI conference websites. Grey literature resources and reference lists of existing systematic reviews were searched to identify relevant studies. For the purpose of this review, “feasibility” is defined as capacity of health system to provide and patient’s accessibility to ART monitoring services following WHO’s recommendations [7].

### Study selection

Studies met inclusion criteria for this review if they: (i) involved HIV infected patients requiring ART and treatment follow-up, and/or healthcare workers involved in providing ART services; (ii) involved a decentralized model of HIV treatment and care which was defined as ART initiation and/or ART monitoring services provided at non-hospital settings: primary health facility or community level (through home-based delivery or community outreach including mobile health services); and (iii) reported one or more of the primary outcomes of interest as defined below.

1. Proportion of patients receiving (with data documented) CD4 count, clinical HIV staging, and/or HIV viral load monitoring at treatment follow-up at regular intervals (6 or 12 months);
2. Proportion of patients receiving ARV drug toxicity monitoring (clinical and/or biomedical) at treatment follow-up at regular intervals; and/or
3. Reported enablers, barriers and other implementing issues related to monitoring of ART services, including any of the following (a) human resources (availability and quality of clinical staff; staff competency training); (b) availability of, and access to, clinical, biochemical monitoring tools for monitoring treatment response, diagnosing ARV drug toxicity, and/or treatment failure; (c) supply chain management: reagents, equipment maintenance, etc. under decentralized HIV care; (d) patient and provider’s attitude towards decentralization of HIV treatment and care.

Secondary outcomes included: (1) Proportion of patients with reported treatment failure, and (2) Proportion of patients who switched to a second line ARV drug.

In order to be eligible for inclusion, studies must have been conducted in LMICs and have reported at least one primary outcome or provided data which allowed for calculation of treatment monitoring uptake.

### Data extraction and data synthesis

Data were extracted electronically using a pre-constructed, standardized data extraction form. Double data extraction with 20% duplication was performed by two independent reviewers. Extracted information included: study details (author/year, objective, design, number of patient enrolled), study population criteria, mode of ART services and outcome of interest. Data on outcomes of interest were grouped, presented and compared by models of service delivery (decentralized vs centralized), time point of treatment follow-up, and study design/study setting context. Quantitative data were presented and analyzed descriptively and data across studies were pooled, provided study interventions and populations were sufficiently similar. Qualitative data were thematically categorized using main themes relevant to the research questions, which emerged from data extracted.

## Results

### Study characteristics

**Fig. 1:**
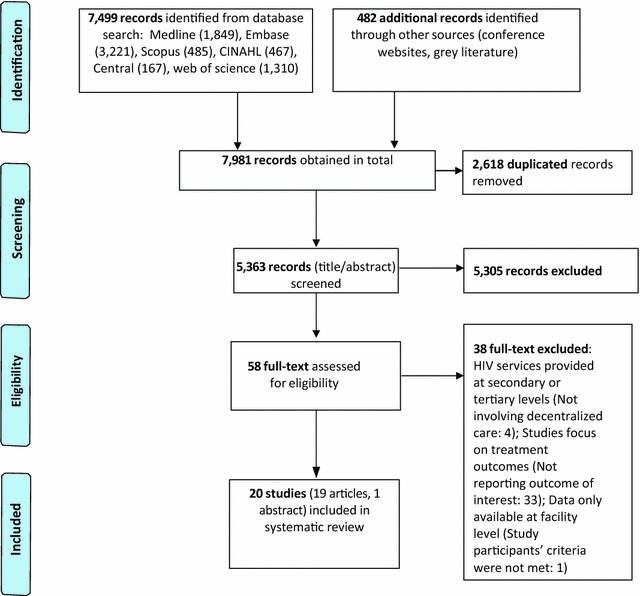
Selection process of included studies

The search strategy identified 5363 titles after duplicates were removed. Screening of titles plus abstracts with exclusion of clearly irrelevant studies resulted in 58 eligible studies for full text review, of which 20 studies (19 articles and one abstract [12]) met all of the inclusion criteria, and were included in the review (Fig. 1).

All but one of these 20 studies were conducted in sub-Saharan Africa (SSA): 10 studies were from various urban, peri-urban and rural settings in South Africa (SA), one study was from rural and urban Ethiopia, one from rural Lesotho, one from rural and urban Kenya, two from rural Rwanda, one from urban Mozambique, one from rural Zimbabwe, two from rural Swaziland, and one from Asia (Thailand). Only two studies were specifically designed to assess the coverage of HIV treatment monitoring services in a decentralized setting; other studies evaluated and reported treatment outcomes. Only one study reported the proportion of patients who developed drug toxicity and two studies provided qualitative data (Table 1).

**Table 1:** Characteristic of included studies

First author, year	Study design	Study participant	Study location/country	Study objective	Model of ART services	Additional resources provided	Study duration/period	Number of patient enrolled	Outcomes of interest	
									Primary	Secondary
Assefa, 2011 [30]	Mix method: retrospective cohort and qualitative study	HIV patient started ART at health facilities providing ART services	55 health facilities 25 health centers and 30 hospitals representing different regions of Ethiopia	To evaluate outcomes of ART services at health centers vs hospital	ART services led by health officer, nurses and CHWs at health center	Community health workers: adherence counseling, defaulter tracking referral and linkage between facilities	Sep 2006–Mar 2009	6206 at health centers; 31,929 at hospitals	Proportion of patient with CD4 count documented Stakeholder’s perspectives on new decentralized model of care	
Bedelu, 2007 [17]	Retrospective cohort	Adult HIV patient	Primary care clinics and hospitals/Lusikisiki, South Africa (SA)	To assess the effect of decentralization and task-shifting on treatment outcomes	ART service led by nurses at health clinic	MSF supported through mobile team visit, training/mentoring of nurse; engage community through support groups	Apr 2004–Apr 2006	1025 (595 at clinic; 430 at hospital)	Proportion of patient with CD4 count and VR data documented	
Boulle, 2010 [19]	Retrospective cohort	Adult treatment naïve HIV patient ≥14 years old	HIV treatment program at three public primary care clinics (PHC) Khayelitsha, SA	To describe outcomes of ART program for adult up to 5 years	Nurse based care with stable patients seen by a nurse 2–3 monthly	Program established/supported by MSF	2001–2007	7323	Proportion of patient with CD4 count and viral load data available	Proportion of patient reported treatment failure; % patient switch to 2nd line ART
Brennan, 2011 [16]	Retrospective cohort	ART naive patient >18 years old	Urban HIV clinic and local PHC in Johannesburg SA	To compare one year outcomes between patient down-referred and maintained at central clinic	ART initiated at hospital and then follow-up at PHC by nurse	Nurse received down-reference training; supervise by doctor, plus advice by electronic treatment algorithm	Apr 2004–Sep 2008	2772	Proportion of patient with 12 month CD4 and VL available	
Fatti, 2010 [41]	Retrospective cohort	ART naïve adult patient >16 years	59 public facilities: 47 PHCs, nine district and three regional hospitals in four provinces of SA	To compare treatment outcomes at different levels of health system (primary health care, district and regional hospital)	ART services led by doctor at different levels	Community-based adherence counselor; all sites supported by NGO (absolute return to kids) with free services to HIV patients	Dec 2004–Dec 2007	29,203	Proportion of patient with viral load results	
Fairall, 2012 [13]	Randomized control trial	Adult patient who had received ART for at least 6 months and were on ART at time of enrollment	31 clinics (16 intervention and 15 control) in free state of SA	To assess effects of task-shifting program on treatment outcomes	ART service led by nurse at primary care clinics (intervention) vs doctor at hospital OPC (control)	Outreach training for nurses with doctor support	Jan 2008–Jun 2010	3029 (intervention) 3202 (control)	Proportion of patient with VR data available	
Humphreys, 2010 [27]	Prospective cohort	Adult patient on ART at least 4 weeks CD4 >100	Primary care clinics and district hospital in rural Swaziland	To assess effect of nurse led primary care based ART program	ART service led by nurse at primary care clinics vs doctor at hospital	Training for primary care nurses by hospital followed by monthly outreach support visits	Jan–Nov 2007	474	Patient experience with primary based ART program	
Hansudewe-chakul, 2012 [15]	Retrospective cohort	HIV infected children	Tertiary hospital and community hospital in rural Thailand	To assess effects of decentralization of pediatric HIV care model	ART initiated at tertiary hospital, monitored at community hospital	Training and mentoring for CH staffs; trained PLHIV: adherence, psychological support	Feb 2002–Mar 2008	410	Proportion of patient with VL data recorded	
Janssen, 2010 [21]	Prospective cohort	HIV patient <15 years on ART	Primary care clinics, KwaZulu-Natal, SA	To assess clinical outcomes of children in a decentralized model	Nurse/counselor led ART program	Home-based care program with nurse/community volunteer providing first aids, nutrition, adherence support at home	Jun 2004–Jun 2008	477	Proportion of patients receiving CD4 and VL monitoring	
Jobanputra, 2014 [26]	Retrospective cohort	HIV patient on ART	Primary health care clinics in rural poor Shiselweni region of Swaziland	To assess program quality, cost and outcomes of routine VL monitoring	Nurse led ART program	MSF support (laboratory equipment, reagent, training of staff)	Oct 2012–Mar 2013	5563	Proportion of patients receiving routine VL monitoring	Reported treatment failure rate; % patient switch to 2nd line ART
Labhardt, 2012 [29]	Retrospective cohort	HIV patient >16 years old on ART with at least three drugs	Two hospital and 12 health centers of Botha-Bothe ad Thaba-Tseka districts of Lesotho	To assess the effectiveness of decentralized ART program	Nurse led ART program	ART program supported by a Swiss NGO through the SolidarMed ART project	Jan 2008–Apr 2011	3747	Availability of treatment monitoring tools at decentralized settings	
Mutevedzi, 2010 [20]	Retrospective cohort	Adult patient >16 years old	16 primary care clinic in rural SA	To describe and assess scale-up of decentralized HIV treatment program	ART initiated by doctor and monitored by nurse	Support for program provided by PEPFAR	Oct 2004–Sep 2008	3010	Proportion of patient with VL data recorded	
Rich, 2012 [22]	Retrospective cohort	HIV patient on ART	ART clinics at health centers in rural Rwanda	To assess clinical outcomes of HIV treatment program	Community-based ART program with directly observed ART and psychosocial supported provided by CHWs	Ongoing HIV education, nutritional assistance, travel allowance for clinic visits, diagnosis and treatment of TB; additional doctor/provider support	Jun 2005–Apr 2006	1041	Proportion of patient with CD4 and VL monitoring data available	Proportion of patient change treatment regimen due to toxicity; % patient switch to 2nd line ART
Selke, 2010 [14]	Randomized control trial	HIV patient, 18 years stable on ART at least 3 months	HIV clinic in rural health center of Kenya	To assess impact of task shifting	Nurse led ART service with home based visit by community care coordinator (CCCs) vs standard of care (no CCCs)	Electronic device support tool (PDA) for patient monitoring; program supported by USAID	Mar 2006–Apr 2008	208 (96 intervention; 112 control)	Proportion of patient monitored with clinical, immunological, virological data	
Shumbusho, 2009 [24]	Retrospective cohort	HIV treatment-naïve adult patients	Three rural primary health centers in Rwanda	To evaluate results of pilot task-shifting model for ART service provision	Nurse centered ART services (initiation management and referral of complex cases	Additional personnel provided for intervention (specific number not reported)	Sep 2005–Mar 2008	1076 (641 pre-ART and 435 on ART)	Proportion of patient with CD4 count documented	Proportion of patient change treatment regimen due to toxicity; % patient switch to 2nd line ART
Sanne, 2010 [25]	Randomized control trial	Adult HIV-1patient (>16 year old, CD4 <350 or previous AIDS defining illness; not pregnant)	Two primary health care sites in Cape town and Johannesbur, SA	To compare outcomes of nurse vs doctor management of ART	ART services led by nurses (vs doctor led): full decentralization	Not reported	Feb 2005–Jan 2009	812	Proportion of patient reported drug toxicity	
Uzodike, 2015 [18]	Cross-sectional	Adult HIV patient on ART	Primary healthcare (PHC) clinics in Kwazulu-Natal, SA	To assess monitoring and referral of patient on ART managed at PHC clinics	ART services led by nurses	Not reported	Jun 2011–Jun 2012	488	Proportion of patients with CD4 VL monitoring data available	% patient reported virological failure
Vogt, 2015 [23]	Retrospective cohort	HIV patient >18 years old initiated on ART at district hospital and rural health clinics (RHCs)	Beitbridge district hospital and six RHCs in Matabeleland South province, Zimbabwe	To compare coverage of CD4 testing between rural and urban HIV patient during 1st year of treatment	HIV care services provided by nurses at RHCs through weekly outreach visits	Services provision supported by MSF (MSF nurse and phlebotomy equipment)	Jan 2011–Dec 2012	2145	Proportion of patients receiving CD4 testing	
Walter, 2014 [12]	Before–after (decentralization) comparison	HIV adult patients initiated on ART at primary health centers		To compare treatment outcomes before and after decentralization	ART service led by nurses at primary health care center	Not reported	2003–2006 (before) 2009–2011 (after)	3936 (before); 13,505 (after)	Proportion of patient with CD4 count documented	
Georgeu, 2012 [28]	Qualitative	HIV patient, service providers (physician, nurse)	Primary health care clinics in free state of SA	To explore experience, perceptions of various stakeholders on implementation process of decentralization of ART services	ART service led by nurses	Not reported	Oct 2007–Jun 2008	16 FGDs, 26 in-depth and key informant interview	Implementing issues related to decentralization Stakeholder’s perspective on decentralization	

### HIV viral load (VL) monitoring

Twelve studies (Table 2) provided data regarding the proportion of patients who received regular VL monitoring, among which 11 studies reported the proportion of patients receiving VL monitoring at 12 months follow-up, with a median service uptake of 86%. The highest coverage of virological monitoring services was reported from two randomized control trials (RCT) conducted in SA [13] and Kenya [14] with 92% (2582/2823) and 99% (86/87) uptake; both studies were conducted by dedicated research staff who were not part of the routine clinical service. The lowest reported proportion of patients with VL monitoring data came from a retrospective cohort study conducted between 2002 and 2008 in rural Thailand [15] with only 14.3% (22/154) of patients having VL data available at baseline and at least one treatment follow-up 12–48 months after treatment initiation. The authors reported that routine VL testing was not available, baseline VL data were available only for a subset of the study participants and VL was determined at 12 months intervals during the 48 months of study.

**Table 2:** Uptake of regular monitoring for patients on ART at different time points of treatment follow-up

First author, year	Follow-ups	Number of patient retained in care	Proportion of patient monitored for treatment response n (%) by # monitoring approaches			Laboratory testing services	Testing site	Notes
			Virological	Immunological	Clinical^a^			
Assefa, 2011 [30]	6 months	Health center: 5072/6197 Hospital: 24,821/31,269	Not report (NR)	54%	NR	Not stated	Not stated	Number of patients received immunological monitoring with CD4 count documented was not reported. Data (proportion of patient monitored) was not reported separately for each level of care
	12 months	Health center: 3042/4022 Hospital: 17,037/23,039	NR	51%				
	24 months	Health center 650/856 Hospital : 4419/6595	NR	51%				
Bedelu, 2007 [17]	12 months	Health clinic: 482/595 Hospital: 289/430	Health clinic: 296/482 (61.4%) Hospital: 41/289 (14.2%)	Health clinic: 348/482 (72.2%) Hospital: 81/289 (28%)	NR	Not stated	Not stated	
Boulle, 2010 [19]	1 year	4512	3932/4512 (87%)	3823/4512 (85%)	NR	CD4 and VL provided 6-monthly after staring ART	Not stated	VL: NucliScens EasyQHIV-1 assay CD4: single-platform panleucogating method Type of blood used not reported
	2 years	2561	2198/2561 (86%)	2108/2561 (82%)				
	3 years	1235	983/1235 (79.6%)	931/1235 (75.4%)				
	4 years	458	351/458 (76.6%)	341/458 (74.5%)				
	5 years	191	148/191 (77.5%)	127/191 (66.5%)				
Brennan, 2011 [16]	12 months	Hospital : 1958/2079 Primary health care: 681/693	Hospital: 1774/1958 (90.6%) PHC 676/681 (99.2%)	PHC: 95% Hospital: 81%	Clinical monitor performed every 2 months by nurse at PHC; 6 monthly by doctor at hospital	CD4 and VL test measured every 6 month	Not stated	Inconsistency in data on % patients with CD4 and VL data available between text and table. Number of patients with 12 months CD4 count available not reported
Fatti, 2010 [41]	12 months	11,960	6725 (56.2%)	NR	Patients attend monthly clinical checks	CD4 count and VL monitored 6 monthly for patient on treatment by SA NHL services	Off-site except for large hospital	Data (n/N) on proportion of patient received and had VL available was reported for all level of care
	24 months	4029	2525 (62.6%)					
	36 months	545	342 (62.8%)					
Fairall, 2012 [13]	12 months	Primary care: 2823/3029 Hospital: 2981/3202	Primary care: 2582/2823 (91.5%) Hospital: 2656/2981 (89.1%)	NR	NR	Not stated	Not stated	
Hansudewe-chakul, 2012 [15]	12, 24, 36, 48 months (VL data available at baseline and at least 1 follow-up)	Community hospital: 154 Tertiary hospital : 133	CH: 22/154 (14.3%) TH: 38/133 (28.6%)	NR	Clinical monitoring using CDC classification;	CD4% assessed 6 monthly, routine VL testing not available. VL measured at 12 month intervals for 48 months	Not stated	No time point specific provided for % patient with VL data available. Scheduled clinic visit 6 monthly at tertiary hospital
Janssen, 2010 [21]	6–12 months	447	193/447 (43.2%)	CD4%: 310/447 (69.3%); CD4: 315/447 (70.5%)	NR	Laboratory tests (CD4, VL, Hemoglobin/albumin) repeated 6 monthly	VL testing at referral hospital (75 km away); other tests at local hospital	
Jobanputra, 2014 [26]	12 months	5563	4767/5563 (86%)	NR	NR	VL measured annually using a Generic HIV VL platform (Biocentric)	VL testing at regional virology laboratory	
Mutevedzi, 2010 [20]	12 months	2527/3010	758/2527 (30%)	NR	NR	CD4 count and VL measured every 6 months	VL testing at provincial lab	VL measured by Nucli-Sens Easy HIV-1 assay). Type of blood used not report
Rich, 2012 [22]	24 months	926	275/926 (29.7%)	710/926 (76.7%)	NR	CD4 count measured 6 monthly using BD fluorescence-activated cell sorting count system	Not stated	
Selke, 2010 [14]	12 months	Intervention: 87 Control: 102	Intervention: 86/87 (99%) Control: 96/102 (94.1%)	Intervention: 87/87 (100%) Control: 96/102 (94.1%)	Intervention: 74/87 (85%) Control: 87/102 (85.3%)	VL and CD4 count obtained at initial and close out research visit. Additional CD4 count at 6 months	Not stated	
Shumbusho, 2009 [24]	6 months	217	NR	193/217 (88.9%)	83.4%: side effect screening at all visits (frequency not reported)	CD4 count measured every 6 month using BD FACS Count	District hospital laboratory	
	12 months	123	NR	104/123 (84.5%)				
	18 months	43	NR	31/43 (72.1%)				
	24 months	10		10/10 (100%)				
Uzodike, 2015 [18]	Jun 2011	488	407/488 (83.4%)	461/488 (94.5%)	412/488 (84.4%)	CD4 and VL monitored 6 monthly	Not stated	Clinical monitoring carried out monthly by nurses
	Dec 2011		466/488 (95.5%)	464/488 (95.1%)	NR			
	Jun 2012		444/488 (91.0%)	430/488 (88.1%)	381/488 (78%)			
Vogt, 2015 [23]	6 months	1250	NR	194/1250 (15.5%)	1250/2145 (58%)	Whole blood collected in EDTA tube for testing by BD Fascount and Partect Cyflow	District hospital laboratory	Data is presented for both hospital and RHCs
	12 months	1199	NR	74/1199 (6.2%)	1199/2145 (56%)			
Walter, 2014 [12]	6 months	11,243	NR	5859 (52%)	NR	Not stated	Not stated	
	12 months	8644		5160 (60%)				
	18 months	6467		4110 (64%)				
	24 months	4485		3201 (71%)				

^a^Clinical monitoring using WHO clinical staging except mentioned otherwise

In four studies that reported the proportion of patients who received VL monitoring in both centralized and decentralized models of care, two studies reported a higher proportion of monitoring of patients attending centralized care (vs decentralized care): 99% (1774/1958) versus 91% (676/681) [16] and 29% (38/133) versus 14% (22/154) [15], while another two studies reported a similar or higher proportion of patients with access to VL monitoring with decentralized care (vs centralized care): 92% versus 90% [13] and 61% (296/482) versus 14% (41/289) [17]. In the two latter studies, both conducted in SA, the difference in service coverage between models of care was not discussed; however, one study [13] reported results of a 30 month randomized trial aimed to assess the effects of an outreach training program provided to nurses for ART initiation and prescribing at primary care clinics while the other [17] reported outcomes of a community-based, decentralized HIV services delivery program supported by Medecins Sans Frontieres (MSF).

Overall, studies conducted in urban settings reported a higher uptake of VL monitoring services: three studies conducted in urban HIV clinic settings in SA [16, 18, 19] reported more than 80% of patients had VL data available after 6–24 months on treatment while three other studies in rural settings (two in South Africa [20, 21] and one in Rwanda [22]) reported 30–43% of patients had access to this service at 12 months follow-up although almost all (five of six) studies stated that VL (and CD4) was planned to be measured 6 monthly for all patients on ART. The ability of nursing staff to establish virological failure for timely referral and regimen switch was a concern as only 59% of patients who demonstrated persistently elevated VL in two consecutive VL monitoring tests were referred for further treatment intervention [18]. None of the included studies reported on-site VL or CD4 testing.

Among 12 studies that included data regarding virological assessment, only three studies reported the platform used for viral load testing (two studies with Nucli-Scens EasyQ HIV-1 and one study with a generic HIV VL platform-Biocentric) and none of these three studies discussed the blood sample used for VL testing (plasma or dried blood spot). None of the other nine studies reported how and where virological and/or immunological monitoring for patients on treatment was conducted.

### Clinical and immunological monitoring

The majority (15 of 20) of included studies reported the use of WHO clinical staging to assess and monitor patients’ responses to treatment (Table 2). Only three studies specifically provided data regarding the proportion of patients who received clinical monitoring through decentralized HIV treatment programs. The other 12 studies did not provide sufficient data for calculation of the coverage of clinical monitoring at decentralized settings.

One study [18] conducted in urban SA assessed the adherence of nursing staff at a primary health care clinic to national guidelines regarding monitoring and follow-up of HIV patients on ART. In this retrospective, cross-sectional study the authors randomly selected and assessed medical records of 488 patients attending the clinic from June 2011 to June 2012 and reported 84% (412/488) and 78% (381/488) patients with clinical monitoring data available by June 2011 and June 2012 respectively.

Another study [14] aimed to evaluate clinical outcomes of patients enrolled in a community-based HIV care program delivered by PLWHs (intervention group) as compared to patients receiving standard, clinic-based care (control group). The reported proportions of patients monitored clinically at 12 months follow-up were identical for both groups, 85% (74/87) for the control group and 85.3% (87/102) for the intervention group. One study [23] reported a lower level of clinical monitoring coverage with 1250 (58%) and 1199 (56%) out of 2145 patients initiated on ART receiving clinical assessments at 6 and 12 months follow-up, respectively.

Among 11 studies with patient follow-up periods from 6 to 24 months, the reported proportion of patients with a CD4 count measurement with data recorded at 6–12 month intervals ranged from 6 to 100%. One study [19] with follow-up data of up to five years reported that 67% (127/191) to 85% (3823/4512) of patients had their CD4 count measured, and 78% (148/191) to 87% (3932/4512) of patients having their VL measured, at 12 month intervals. Data from this study showed that the proportion of patients receiving immunological and virological monitoring decreased over time, although the total number of patients in care also reduced by 96% after 5 years on treatment (from 4512 after 12 months to 191 after 5 years follow-up).

Two studies provided data that compared coverage of immunological monitoring between decentralized and centralized HIV care sites and both studies reported a higher uptake of services in the decentralized model. One study [17] reported 72% (348/482) of patients attending rural primary health care clinics versus 28% (81/289) attending a hospital had their CD4 count determined after 12 months of treatment. The authors reported that ART services provided at primary clinics were supported by Médecins Sans Frontières (MSF) with involvement of peer support groups to track defaulters, provide adherence support, advocate for better drug supply and monitoring of HIV program whereas no such supports were provided to patients receiving care at hospital. The second study [16] reported 95% of down-referred (decentralized) patients (n = 693) versus 81% of centralized patients (n = 2079) had a CD4 count (and VL) available at 12 months but the information on treatment monitoring procedure (platform used for VL testing, type of blood sample used and place where VL testing performed) was not presented.

One study [23] aimed to assess the coverage of immunological monitoring between HIV patients living in a semi-urban district in Zimbabwe and reported only 21 and 8% of urban (n = 1545), and 2 and 1% of rural patients (n = 600) had received CD4 testing at 6 and 12 months follow-up, respectively. The authors reported that CD4 testing at rural health centers was usually restricted to the day of outreach visits when outreach staff collected blood samples in Ethylenediaminetetraacetic Acid (EDTA) tubes and brought the blood back to the district hospital for testing within 24 h. Limited capacity for specimen transportation within local health systems was noted as the main reason for the differences in access to CD4 testing between rural and urban patients.

### Toxicity monitoring

No study reported the proportion of patients receiving laboratory monitoring for ARV drug toxicity at scheduled monitoring visits in program settings. The proportion of patients who changed their initial regimen (drug substitution, not considered as switching to second line ART) due to drug toxicity was reported as ranging from 5% (161/3029) [13] to 29% (304/1040) [22]. One retrospective cohort study [24] reported 83.4% of all patients were screened for side-effects at all visits but the frequency of visits was not stated. A randomized controlled trial [25] reported 17% (68/404) and 16% (66/408) toxicity failure in patient groups managed by a nurse and by a doctor at primary care settings, respectively.

### Secondary outcomes

Three studies reported the proportion of patients with virological failure and four studies reported the proportion of patients who switched from 1st to 2nd line ART. The reported proportion of patients with treatment failure ranged from 14% (n = 4512) [19] to 49% (n = 488) [18] and the proportion of patients starting 2nd line treatment was from 0.5% (n = 1040) [22] during 24 months follow-up to 12.2% (n = 4512) [19] at 60 months on treatment. One study assessed the outcomes of routine VL monitoring of ART programs through a decentralized network of 22 primary care clinics and three reference facilities in Zimbabwe. These investigators reported 17% (551/3242) of VL tests had detectable HIV (>1000 copies/µL) and among 288 patients with an initial detectable VL result, 78 patients (27%) did not receive adherence counseling, 86 (30%) patients had no follow-up VL, and 15 patients (5.2%) patients were switched to 2nd line treatment, among whom four patients were switched based on a single detectable VL result [26].

### Factors that influence the implementation and feasibility of decentralization

Data from included studies suggest that patients were supportive of decentralization of HIV treatment and care as it could help to improve their access to care (Table 3). One study [27] reported 96% (29/31) of patients interviewed were ‘very satisfied’ or ‘satisfied’ with HIV treatment services provided by nurses, and the main reasons for this included reduced cost, receiving services near home and shorter queue, and being treated better by staff. Health professionals also reported positive responses: nurses were comfortable, motivated, enthusiastic about the opportunity to be directly involved in providing life-saving treatment; physicians supported decentralization and nurse-led ART initiation as it could help increase ART coverage, but expressed uncertainty about the ability of nurses to manage and refer complicated cases [28].

**Table 3:** Influencing factors for monitoring of ART services in decentralized settings

First author, year	Reported implementing issues/barriers for ART services under decentralized care from system perspective	Acceptability and reported quality of decentralized care from service provider and patient perspectives
Assefa, 2012 [30]	Policy: lack of regulation framework enabling nurse to perform tasks such as ARV prescription, monitoring of patients on ART Finance: high cost associated with training and monitoring quality of services Human resource: additional workload for nurse without increased remuneration/compensation; Community health workers were not permanent employee/formal health system	Patient’s perspective: Nurse led ART services was well accepted, help to reduce waiting time; provide appropriate counseling; combat stigma and discrimination in society and can help to provide opportunity for employment
Georgeu, 2012 [28]	Workload, including paperwork increased significantly for nurses and other team members through broader human resource shortage and lack of capacity (e.g. data capturers performed basic nurse duties when nurse too busy, nurse dispensed when pharmacist not available) Increased number of patients on treatment further strained scare/inadequate human and physical resources of health system: insufficiently staff and resources; fragmented information, poor patient transport/referral system; unreliable drug supplies due to poor communication, transport between pharmacy/central dispensing unit and clinics	Nurses were comfortable, motivated, enthusiastic about opportunity to be directly involve in providing life-saving ART treatment Physicians reported mix attitude: majority support decentralization and nurse initiated ART but significant minority reported uncertainty about the ability of nurses to manage and refer complicate cases Patients were supportive of decentralization as it improved access to care, reduced travel time/cost but some wanting ART to remain a separate services and expressed preference toward physician services because of higher clinical status and only doctor can medically certify social grant—key source of income for people living with HIV/AIDS in South Africa
Humphreys, 2010 [27]		Among patients interviewed in intervention group (received nurse led/primary care based ART services) 81% (25/31) were very satisfied 13% (4/31) were satisfied 3% (1/31) dissatisfied and 3% (1/31) very dissatisfied as compared to services at main hospital Reasons for satisfaction includes: reduced cost, services provided nearer to home, shorter queue and being treated better by staff. Reasons for dissatisfaction were lack of doctor and delay of service because team from hospital arrive late
Labhardt, 2012 [29]	CD4, VL and biochemistry were not available on site at decentralized settings Hemoglobin was available in 2/5 and 2/7 health centers of two studied districts	

A number of system factors that could hinder the implementation and scale-up of decentralization in low-resource settings were identified and discussed. These factors include: (i) Limited resources available for treatment monitoring services (ii) Lack of a policy framework which allows non-physician staff (nurses) to initiate HIV treatment; (iii) increased workload (clinical and administrative) for nurses without commensurate remuneration; (iv) unreliable antiretroviral drug supplies due to poor communication, inadequate transport between pharmacy/central dispensing unit and clinics; and (v) high costs associated with health worker training and monitoring of service quality [29, 30].

## Discussion

### Why treatment monitoring is important to achieve the 90-90-90 goal

Monitoring of patients on antiretroviral treatment (ART), especially in the context of rapid scale up of ART coverage in high HIV burden and low-resource settings through different models of services delivery including decentralization, is one of the most important elements to ensure effectiveness and sustainability of any HIV treatment and care program. The “90-90-90” goal aims at having 90% of HIV positive people knowing their infection status; 90% of those people receiving ART, and 90% of those on ART with virologic suppression, and is considered a universal target needed to effectively control and ultimately end the global HIV epidemic. There are two key milestones that need to be achieved to make the last “90” target a reality. First, the majority (>90%) of patients on ART must have access to appropriate and timely ART monitoring: 12-monthly VL assessment or 6-monthly clinical assessment and CD4 count if VL is not available. Second, effective treatment and well-functioning patient support systems including adherence coaching must be in place to achieve a majority (>90%) of patients on ART with sustained viral suppression.

### VL monitoring

WHO has recommended VL testing as the preferred method for monitoring the responses of patients on ART [8] and it has been suggested that in order to achieve the “90-90-90” goal, viral load monitoring should and can become the standard of care in LMICs with high HIV prevalence [31]. There is ample evidence showing that routine VL monitoring can provide an early and more accurate diagnosis of treatment failure when compared to clinical and immunological monitoring [32, 33], but evidence regarding the value of VL monitoring as compared to immunological monitoring in reducing mortality among patients on ART has been mixed [34–38]. Availability of, and access to, VL testing is still limited due to the requirements of expensive laboratory equipment, complex sample collection and processing, and the need for highly trained personnel [39]. A recent WHO survey on availability and use of HIV diagnostics in LMICs found that the overall coverage of VL testing among patients on ART from 94 countries was 23% [40]. In our review, a wide range of VL monitoring coverage was observed. Although we found a median VL monitoring coverage of 86% at 12 months follow-up, this level of coverage may not well reflect regular VL monitoring practice under decentralized HIV care models in LMICs. Among eight studies which reported VL monitoring rate of greater than 50% among patients on ART, five studies reported results of decentralized HIV programs supported by external donors such as MSF [17, 19, 26], Absolute Return for Kids [41], U.S. Agency for International Development (USAID) [14], and two studies reported results of randomized trials in which VL testing was part of the funded studies in well-resourced settings [13, 14]. In addition, existing evidence suggests that the longer a patient is on ART the lower the rate of receiving regular VL test. Only three studies evaluated the long-term (more than 24 months) follow-up of VL coverage [15, 19, 41]. Thus, further research is needed to examine patient retention and treatment monitoring practices with long-term follow-up, particularly in rural settings.

### Clinical and immunological monitoring

At decentralized primary care levels in LMICs, clinical and CD4 count monitoring remains a viable option to monitor treatment responses in settings where VL testing is not available. In our review, limited data were available to assess the feasibility and coverage of clinical and immunological monitoring in a decentralized model of HIV care, as only two studies provided data on the actual proportion of patients who received both clinical and CD4 monitoring. Of note, these are the two studies designed to assess treatment monitoring practice in two different settings, providing a contrasting picture of the coverage of treatment monitoring services. Differences in treatment monitoring coverage between these ART programs could be explained by study settings and service delivery models; one in an urban, well staffed HIV clinics with clinical staff on call and easy access to laboratory testing [18] while the other was in rural areas with ART service provided by outreach teams and with long distances for sample delivery from clinics to a laboratory facility located at a district hospital [23]. This finding highlights an important potential gap in existing knowledge. It has implications related to the implementation of treatment monitoring in future decentralized ART programs, particularly in rural, resource-constraint settings, as when only a minority of patients is engaged in an ART program where they receive regular monitoring, an increase in treatment failure and drug resistance can be foreseen.

### Drug toxicity monitoring

Limited data were available to assess the feasibility of drug toxicity monitoring for patients on ART in decentralized settings. None of the included studies reported the proportion of patients on ART who received laboratory-based drug toxicity monitoring, but one RCT showed that the proportion of patients reporting toxic drug effects (defined as adverse events that required treatment interruption for >42 days) during the study period was higher than the total virological failure rate among patients on ART [25]. This finding is in line with results from other studies suggesting that drug toxicity is the most common reason for changing initial treatment regimen [42, 43]. The WHO guidelines emphasize that laboratory monitoring is not required for treatment initiation. However, there are major toxicities associated with ARV drugs that should be monitored in all patients on treatment. The basic monitoring for potential toxicity of drugs such as tenofovir, zidovudine and nevirapine require laboratory assessment of renal function, hemoglobin, and liver enzymes, respectively. Without the availability of, and access to, these basic tests, monitoring for ART toxicity cannot be performed, and could compromise the long-term effectiveness and sustainability of the ART program. Researches have showed that HIV patients on ART who have regimen substitution due to drug toxicity/drug related adverse reactions were at higher risk of loss to follow-up [44, 45] which may partly explain the significant reduction in number of patients in care after five years of follow-up reported from study included in this review [19].

### Barriers related to treatment monitoring and evaluation of treatment monitoring under decentralization

#### Technological constraints

From a technology perspective, in the absence of point of care (POC) testing, access to laboratory monitoring for patients on ART under a decentralized model of care in low-resource settings will likely be limited. For VL monitoring; until the arrival of a true point of care VL test, the feasibility of VL monitoring for patients received ART at the primary clinic level will depend on system capacity to collect and process a blood sample, transport the sample to a central laboratory for testing and return the result in a timely manner. For immunological monitoring, there are CD4 POC technologies available that can be operated in decentralized settings and produce reliable results for treatment monitoring [46]; their use has been shown to improve access to this alternative monitoring method and increases patient retention along the HIV treatment cascade compared to conventional laboratory testing [47].

#### Human resource constraints

A lack of trained medical doctors for initiation and management of patients on ART has been identified as a major barrier for scaling up of ART programs [48, 49]. Task-shifting of HIV services from physician to non-physician carers has been introduced to overcome this challenge [4, 13]. From a treatment monitoring perspective, however, task-shifting does not come without challenges. Findings from our review suggest that increased clinical and administrative responsibilities associated with provision of nurse-led ART services could further burden the already-limited personnel at primary health care level. Primary health care staff reported their reluctance to put more PLWHA on treatment because of concern over their capacity to manage the burden of an increasing number of patients on ART. The quality of treatment monitoring could also be a concern as nursing staff were unable to identify and refer all cases of treatment failure at decentralized settings for regimen change, even with the availability of two consecutive VL monitoring results indicating virological failure. The lower than expected rate of patients initiated on second line ART may represent an appropriate strategy to optimize adherence before switching therapy but it may also indicate clinicians’ lack of confidence regarding interpretation of VL results and second line treatment. The introduction of any new assay into a clinical setting requires education of the clinician in its interpretation; this is especially the case with a complicated tool such as a VL test. On the other hand, early switching to second line ART after a single detectable VL test without appropriately addressing non-adherence issues would potentially result in the unnecessary initiation of second line ART and, without addressing poor adherence would lead to suboptimal second-line outcomes [50, 51]. This is an important issue of concern particularly in settings where treatment options are limited and second and third-line regimens are costly.

#### Recommendations

**Fig. 2:**
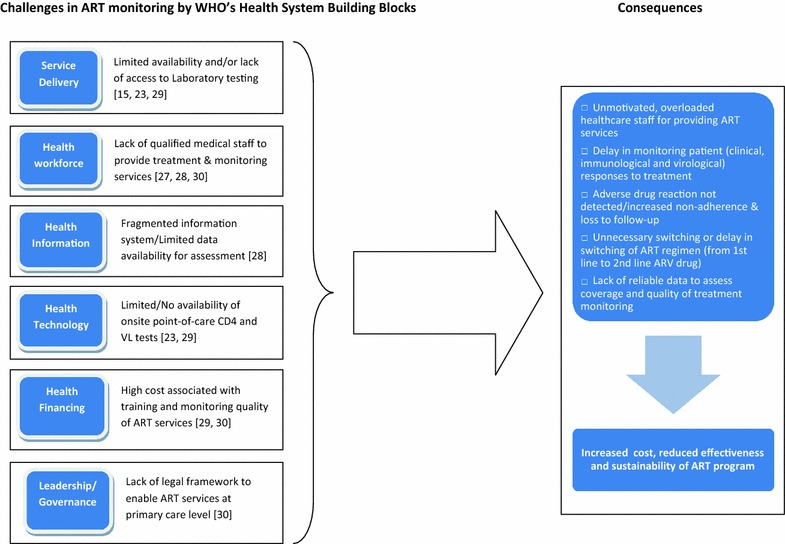
Health system challenges impacting access to ART monitoring (clinical/immunological, viral load and drug toxicity) based on data from studies included in the review

In LMICs the challenge of limited coverage of, and access to, treatment monitoring services that is associated with decentralization of HIV treatment and care often lies within the health care system; therefore a comprehensive strategy to improve the practice of treatment monitoring should be considered from a health system strengthening perspective (Fig. 2).

In terms of service delivery, treatment and treatment monitoring services should ideally be delivered close to where the patient lives, with appropriate diagnostic technology and human resource availability at the primary health care level. The development and implementation of POC technologies to provide immunological and virological monitoring are critically important to ensure appropriate treatment monitoring, particularly with further scale up of HIV treatment services in decentralized settings.

The impact of future studies towards improving the implementation of decentralized care would benefit from the inclusion of some standardized targets and outcomes in published reports. In the absence of clearly defined indicators and targets, the assessment and appraisal of coverage and quality of treatment monitoring services continues to be a challenge. Given the momentum in scaling up ART and towards achieving the 90-90-90 target, there is a need for standardized measures that can be used in many upcoming researches reporting global progress towards this ambitious goal. The development and adoption of a specific set of processes and target indicators regarding treatment monitoring could help to align the reporting system within different levels of health services provision, improve the timeliness of reporting results, and ensure that appropriate action is taken when results support particular interventions (e.g. adherence counseling).

Lastly, from governance and financing perspectives, it is obvious that if the ambitious “90-90-90” goal is to be achieved in 2020, the importance of treatment monitoring must be emphasized equally with the importance of treatment coverage. Substantial resources are required to ensure appropriate treatment monitoring for all people on ART. Critical to success is the assessment of system capacity, particularly human resources and health technology in delivering treatment monitoring. This must be conducted as an integrated component of the decision-making process in order to identify the optimal strategy to increase high quality coverage of HIV treatment and care services in any given specific setting. Expansion of ART coverage without considering system capacity for the provision of appropriate treatment monitoring to all patients will inevitably lead to more treatment failures and increased development of drug resistance, with resulting public health costs to address these problems. Therefore, the recommendation of WHO that lack of access to, or availability of, laboratory monitoring should not be a barrier in initiating patients on treatment may need to be revisited, as the closer we get to the second “90” goal of having 90% people diagnosed with HIV on treatment, the higher the importance of assuring that those patients who are on treatment are also appropriately monitored, such that the last “90” goal of having 90% people on treatment with viral suppression can be achieved.

## Limitation

This review has some limitations that should be taken into account when interpreting the findings. Here, we identified only two studies that aimed to assess the monitoring and management of HIV patients. This paucity of data results in challenges regarding data interpretation and meant that we were unable to analyze and discuss differences in coverage of treatment monitoring services as well as quality of the services. Lack of information and data from unpublished government and program reports and studies published in non-English language may contribute to limited data availability. Moreover, limited data from studies conducted in SSA countries has made it difficult to generalize the findings outside the sub-Saharan African context.

## Conclusions

The findings of this review suggest that there are potential major gaps in coverage and quality of treatment monitoring services for HIV patients on ART. Further studies particularly from non-SSA countries with longer-term of follow up are in need to assess the feasibility of treatment monitoring in the context of decentralization HIV treatment and care in LMICs. Significant investment in POC testing and, improving quality of and training for nursing staff to effectively manage patients on ART is required to improve quality of HIV treatment and care services. The development of a set of target program indicators for treatment monitoring is necessary to reinforce the importance of treatment monitoring in the HIV continuum of care toward achievement of the 90-90-90 goal by the year 2020.

## Additional file

**Additional file 1: Annex S1.** Search strategy: Decentralization of HIV treatment and care in low and middle income countries.

## Abbreviations

ART: antiretroviral therapy
PLWH: people living with HIV/AIDS
LMICs: low and middle income countries
SSA: sub-Saharan Africa
SA: South Africa
VL: viral load
RCT: randomized control trial
POC: point of care
