# Supplementary material for: Feasibility of antiretroviral treatment monitoring in the era of decentralized HIV care: a systematic review
Source: AIDS Res Ther. 2017 Jan 19;14:3. doi: 10.1186/s12981-017-0131-5 (PMC5248527; doi:10.1186/s12981-017-0131-5)
Supplement: Supplementary file 1 — Additional file 1: Annex S1. Search strategy: Decentralization of HIV treatment and care in low and middle income countries. [file 12981_2017_131_MOESM1_ESM.docx]

| **#** | **Searches** | **Return** |
| --- | --- | --- |
| 1 | Antiretroviral Therapy, Highly Active/ or exp Anti-Retroviral Agents/ or exp Anti-hiv Agents/ | 80137 |
| 2 | (HAART or ART).tw. | 59672 |
| 3 | (anti-retroviral or antiretroviral or anti-HIV or antiHIV).tw. | 52876 |
| 4 | 1 or 2 or 3 | 138290 |
| 5 | exp HIV/ or exp HIV Infections/ or HIV Long-Term Survivors/ | 281594 |
| 6 | (human immun* deficiency or human immun?deficiency or acquired immun* deficiency or acquired immun?deficiency).tw. | 85421 |
| 7 | HIV*.tw. | 241449 |
| 8 | 5 or 6 or 7 | 330992 |
| 9 | 4 and 8 | 85651 |
| 10 | exp HIV Infections/dt or exp HIV/de | 70529 |
| 11 | Antiretroviral Therapy, Highly Active/ or exp Anti-HIV Agents/ or HAART.tw. | 70383 |
| 12 | 10 or 11 | 92326 |
| 13 | 9 or 12 | 106599 |
| 14 | Developing Countries/ | 67982 |
| 15 | (Afghanistan* or Albania* or Algeria* or Angola* or Argentina* or Armenia* or Azerbaijan* or Bangladesh* or Belarus* or Beliz* or Benin* or Bhutan* or Bolivia* or Bosnia* or Herzegovin* or Botswan* or Brazil* or Bulgaria* or Burkina* or Burundi* or Cabo Verde* or Cape Verde* or Cambodia* or Cameroon* or Central African or Chad* or China or Chinese or Colombia* or Comor* or Congo* or Costa Rica* or Cote d'Ivoir* or Ivory Coast or Cuba* or Djibouti* or Dominica* or Ecuador* or Egypt* or El Salvador* or Eritrea* or Ethiopia* or Fiji* or Gabon* or Gambia* or Georgia* or Ghana* or Grenad* or Guatemala* or Guinea* or Guyan* or Haiti* or Hondura* or Hungar* or India* or Indonesia* or Iran* or Iraq* or Jamaica* or Jordan* or Kazakhstan* or Kenya* or Kiribati* or Korea* or Kosov* or Kyrgyz Republic or Lao* or Leban* or Lesotho* or Liberia* or Libya* or Macedonia* or Madagascar* or Malawi* or Malaysia* or Maldiv* or Mali* or Marshall Island* or Mauritania* or Mauriti* or Mexic* or Micronesia* or Moldova* or Mongolia* or Montenegr* or Morocc* or Mozambi* or Myanma* or Burmese or Namibia* or Nepal* or Nicaragua* or Niger* or Nigeria* or Pakistan* or Palau* or Panama* or Papua New Guinea* or Paraguay* or Peru* or Philippines or Filipino or Romania* or Rwanda* or Samoa* or Sao Tome* or Senegal* or Serbia* or Seychell* or Sierra Leon* or Solomon Island* or Somalia* or South Africa* or Sudan* or Sri Lanka* or St Lucia* or St Vincent or Grenadines or Surinam* or Swazi* or Syria* or Tajikistan* or Tanzania* or Thai* or Timor* or Togo* or Tonga* or Tunisia* or Turk* or Turkmenistan* or Tuvalu* or Uganda* or Ukrain* or Uzbekistan* or Vanuatu* or Venezuela* or Vietnam* or West Bank or Gaza or Yemen* or Zambia* or Zimbabwe*).mp. | 1803465 |
| 16 | exp africa/ or exp caribbean region/ or exp central america/ or latin america/ or exp south america/ or asia/ or exp asia, central/ or exp asia, southeastern/ or exp asia, western/ or exp indian ocean islands/ or pacific islands/ or exp melanesia/ or exp micronesia/ or exp west indies/ | 649014 |
| 17 | (africa* or asia* or caribbean or central america* or latin america* or south america* or melanesia* or micronesia* or polynesia*).tw. | 249386 |
| 18 | (resource-limited or resource-poor or low-resource* or limited-resource* or resource-constrained or under-resourced or poorly-resourced or resource-scarce or scarce-resource* or low-income or middle-income or LMIC).tw. | 39126 |
| 19 | ((developing or underdeveloped or under-developed or emerging or less-developed or least-developed or less-economically developed or least-economically developed or less-affluent or least-affluent) adj (country or countries or nation or nations or economy or economies)).tw. | 43335 |
| 20 | ((developing or underdeveloped or under-developed or less-developed or least-developed) adj world).tw. | 6206 |
| 21 | (third-world* or 3rd-world*).tw. | 2957 |
| 22 | 14 or 15 or 16 or 17 or 18 or 19 or 20 or 21 | 2069703 |
| 23 | 13 and 22 | 22649 |
| 24 | "delivery of health care"/ or health services accessibility/ | 123680 |
| 25 | Health Facilities/ or Primary Health Care/ or House Calls/ or Primary Care Nursing/ | 73813 |
| 26 | "Health Services Needs and Demand"/ or "Referral and Consultation"/ | 98963 |
| 27 | community health services/ or community health nursing/ or community networks/ or home care services/ or home health nursing/ or home nursing/ | 82682 |
| 28 | rural health services/ or rural nursing/ or Rural Health/ or Rural Population/ | 73000 |
| 29 | Ambulatory Care Facilities/ or Ambulatory Care/ or community health centers/ or mobile clinics.mp. [mp=title, abstract, original title, name of substance word, subject heading word, keyword heading word, protocol supplementary concept word, rare disease supplementary concept word, unique identifier] | 56464 |
| 30 | ((delivery or access*) adj3 (health or healthcare)).tw. | 31252 |
| 31 | (primary health or community health or rural health or primary care or health facilit*).tw. | 107399 |
| 32 | (home-based or community-based or down-refer* or treatment centre* or treatment center* or health center* or health centre* or nurse-based or nurse-initiat* or nurse-manage* or nurse-led or nurse-cent*).tw. | 71396 |
| 33 | (remote area* or district level* or rural setting* or remote setting* or rural facilit* or rural clinic* or rural district* or rural sub-district* or health facilit* or home therapy or home treatment or primary healthcare or peripheral health or peripheral healthcare or community care or community site* or community setting* or home setting*).tw. | 29554 |
| 34 | (decentral* or de-central* or task-shift*).tw. | 5435 |
| 35 | 24 or 25 or 26 or 27 or 28 or 29 or 30 or 31 or 32 or 33 or 34 | 565707 |
| 36 | 23 and 35 | 3935 |
| 37 | "outcome assessment (health care)"/ or treatment outcome/ or treatment failure/ | 792973 |
| 38 | (outcome* or monitor*).mp. | 2130044 |
| 39 | chemically-induced disorders/ or exp "drug-related side effects and adverse reactions"/ or poisoning/ | 114990 |
| 40 | (side effect* or toxic* or adverse reaction* or adverse event* or adverse effect* or poison*).tw. | 818253 |
| 41 | exp CD4 Lymphocyte Count/ or exp CD4-Positive T-Lymphocytes/ or Antigens, CD4/ or CD4.tw. | 171522 |
| 42 | (T4 adj3 (cell* or lymphocyte*)).tw. | 2287 |
| 43 | Viral Load/ or (virus titer or viral load or viral burden).tw. | 35062 |
| 44 | exp Hemoglobins/ or (haemoglob* or hemoglob*).tw. | 167855 |
| 45 | liver enzyme*.mp. or Liver/en | 86032 |
| 46 | Creatinine/ or Urea/ or (urea* or creatinine*).tw. | 175304 |
| 47 | Antiretroviral Therapy, Highly Active/ae [Adverse Effects] | 2957 |
| 48 | exp Anti-HIV Agents/ae, po, to [Adverse Effects, Poisoning, Toxicity] | 10439 |
| 49 | 37 or 38 or 39 or 40 or 41 or 42 or 43 or 44 or 45 or 46 or 47 or 48 | 3351904 |
| 50 | 36 and 49 | 1894 |
| 51 | limit 50 to (english language and yr="1996 -Current") | 1849 |
